# Dispersion is essential in crop residue application

**DOI:** 10.12688/f1000research.16748.4

**Published:** 2025-12-16

**Authors:** Masato Oda

**Affiliations:** 1 Crop, Livestock and Environment Division, Japan International Research Center for Agricultural Sciences, Tsukuba, Japan

**Keywords:** Conservation agriculture, Crop residue, Green manure, Marshall Islands, Organic matter application, Soil degradation, Anaerobic decomposition

## Abstract

**Background:**

Crop residue application can maintain soil fertility and sustain agriculture. However, the effects of residue application for crop growth are unstable because of variable weather conditions and the residual effects of crop residue application. Furthermore, residue application often reduces crop yields. Therefore, I tried to clarify effective residue application factors in an environment which was has stable weather conditions and low residualafter effects.

**Methods:**

Majuro atoll, a coral sand atoll near the equator, was selected for the experiment site because of its stable weather and low residualafter effect of coral sand. A factorial design experiment using sweet corn was conducted based on the following four factors: fungi propagation before application, cutting residue into pieces, dispersion (or accumulationwindrowing) of applied residue, and placement (on the surface or incorporation) with an equal amount of crop residue. The effects of each factors on the corn yields were evaluated using Cohen’s effect size analysis.

**Results:**

The dispersion showed the largest effect (1.2 in Cohen’s), which exceeded the effect of incorporation (0.7). The interaction of dispersion and incorporation showed a huge effect (4.9) on corn yield.

**Discussion:**

The effect of dispersion was not positive but it avoided the negative effects of residue clustering. Because, the toxicity of the plant residue and generation of toxic substances by anaerobic decomposition are widely known. Anaerobic decomposition occurs inside the residue clusters. However, dispersion reduced the toxicity by adsorption in soil and avoiding anaerobic decomposition. Furthermore, incorporation showed an interaction effect, but surface placement did not.

**Conclusion:**

The dispersion of crop residue enhanced the positive effect of crop residue incorporation by avoiding the toxicity from crop residue. This finding adds a new viewpoint for the controversy between conventional and conservation agriculture.

## Introduction

Soil degradation is a major constraint on food security ( Gomiero, 2016; Lal, 2015), and the intervention points for reversing soil degradation are soil erosion and depletion of soil organic matter ( Karlen & Rice, 2015). Conservation agriculture or green manure approaches are developing against this backdrop and emphasize the importance of retaining crop residue ( Cherr *et al*., 2006; Pittelkow *et al*., 2015a); however, they have not yielded satisfactory results. For example, long-term trials of conservation agriculture show no particular interactions among factors such as tillage, mulch, rotation, soil texture and rainfall ( Pittelkow *et al*., 2015b).

The main problem with crop residue application is that the decomposition of the residue can have both positive and negative effects on crop production ( Kumar & Goh, 1999). Nitrogen benefits and nitrogen recovery from residues show that they have considerable potential ( Kumar & Goh, 1999). However, microorganisms produce toxic substances during the decomposition of plant residue. Microorganisms metabolize these substances in anaerobic soils. Nonetheless, the phytotoxic leachates of some decomposing cover crop residues have adverse effects on crops even under aerobic conditions ( Bhowmik & Doll, 1982; Jessop & Stewart, 1983; Kimber, 1973a; Kimber, 1973b; Lynch, 1977; Lynch, 1978; Stirzaker & Bunn, 1996).

The application method of crop residue is a major factor in the success of the method, although the effects of organic matter application also differ by the quantity, conditions and timing ( Chen *et al*., 2014; Tian *et al*., 2007). Both particle size and placement of the applied material affect the residue breakdown rate and the mineralization/immobilization process ( Kumar & Goh, 1999). However, environmental factors have strong interactions with residue decomposition ( Kumar & Goh, 1999). Finding effective crop residue application methods is difficult without stable weather and with the inability to control for no after effects of organic matter, even with a focus on the application method.

The aim of this study was to clarify the most effective crop residue application method. Although, there are many factors concerning residue management ( Kumar & Goh, 1999), I examined four factors (fungi propagation, cutting, dispersion and incorporation) from the viewpoint of farmers applicability. Experiments were conducted in low fertility and high microorganism’s activity environment. That enabled clear observing the effect of applied residue on crop yields.

## Methods

### Site description and conditions

Majuro Atoll, the capital of the Republic of the Marshall Islands, is located in the Pacific Ocean near the equator, where year-round cultivation is possible. The maximum and minimum monthly average temperatures are 29.4 to 30.2°C and 24.7 to 25.0°C, respectively. The average monthly precipitation is 169 to 356 mm, and the average annual precipitation is 3, 365 mm ( CLIMATE-DATA.ORG). Coral sand, the soil of Majuro atoll, has relatively high organic matter levels in top soil due to refractory organic matter (organic carbon of 46.9 g kg ^−1^ at 0–15 cm and 10.8 g kg ^−1^ at 15–45 cm) ( Deenik & Yost, 2006), and high percolation rates (1.4–3.5 × 10 ^−3^ m s ^−1^) ( Hunt & Peterson, 1980). Thus, the climate is stable, the soil has high permeability with no waterlogging, and frequent rainfall keeps the soil moisture close to field capacity, so applied organic matter is almost decomposed in the crop period. It is known that the after effect of organic resources is small for sandy soils in a warm and humid climate ( Chivenge *et al*., 2011).

The experimental field was located at Laura Farm (7°8"34"N, 171°2"9"E), which belongs to the Ministry of Resources and Development. The field size was 12.5 × 7.2 m. The plot size was 1.2 × 6.0 m, and the experimental field consisted of two sets of six plots. The government forbids the use of synthetic fertilizer and chemicals on this atoll in order to protect the underground aquifer; therefore, farmers use copra cake as fertilizer. Soil water-soluble NO _3_-N (0–5 cm layer; 1:2.5 soil:water extraction) was 4 µg g soil ^−1^, as assessed by a nitrate ion meter (LAQUAtwin B-742, Horiba, Tokyo). No significant after effect has been found in this field, although corn had been cropped three crops without fertilizer nor any chemicals from November 26, 2013 to July 28, 2014 (see raw data ( Oda, 2018)).

### Materials and crop management

Corn residue to be used as the material for the experiment was harvested on July 28, 2014, just after the harvest of the previous crop of corn in the experimental field. The residue was divided into 12 bundles of the same fresh weights of 4.7 Mg ha ^−1^ (2.1 Mg ha ^−1^ in dry weight) and applied to the plots by different methods (described later). Corn ( *Zea mays* L.) was planted in two rows at 0.5 m row spacing at a population of 3.3 plants m ^−2^ on August 4, 2014, and harvested on October 21, 2014 (78 days after seeding). Irrigation, fertilizer and chemical applications were not performed. Hand weeding was performed 2 and 5 weeks after seeding. Weed emergence was negligible and the weed residue was left on the soil surface of the plot.

### Treatments

**Figure 1.**
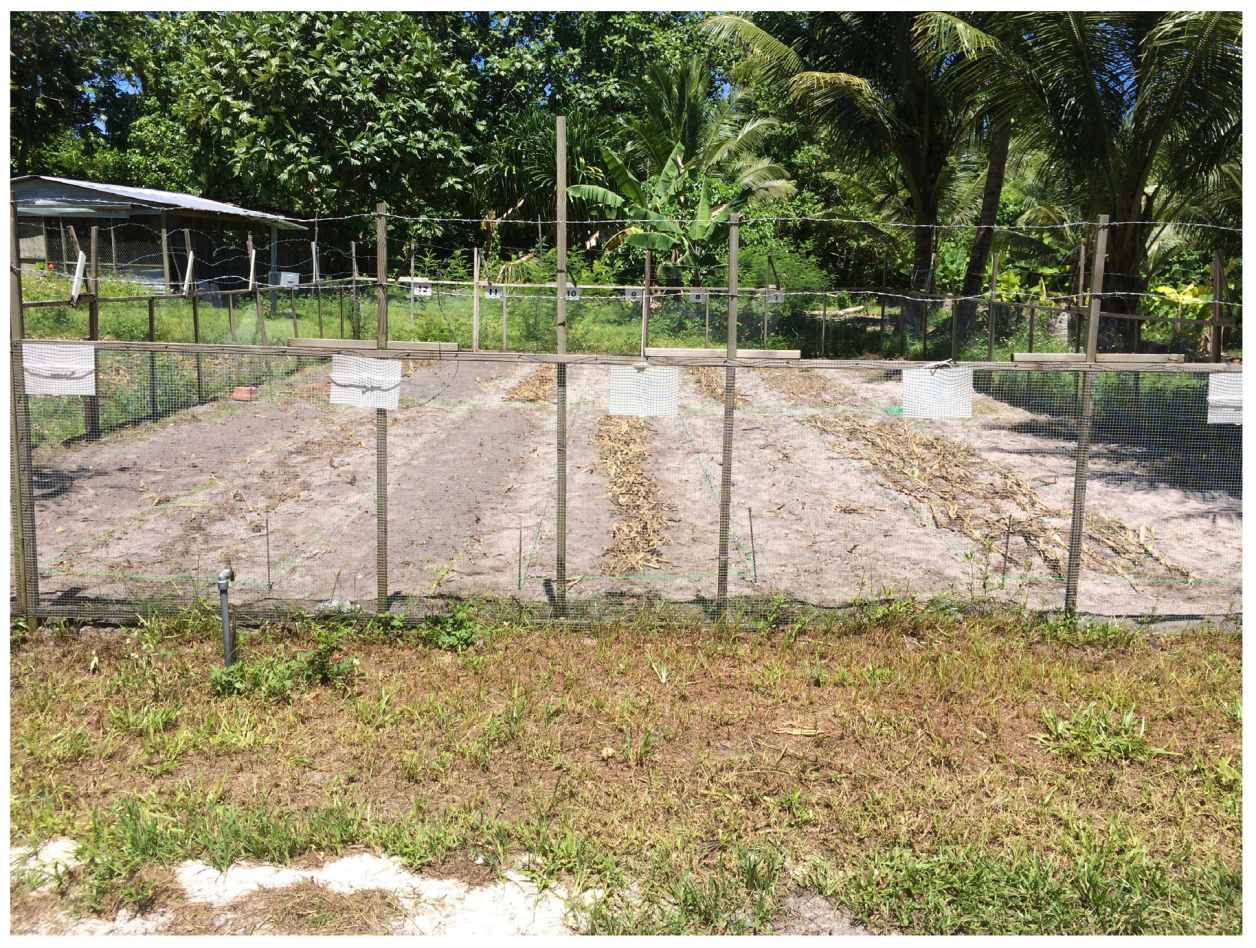
Photograph of the condition of the experimental field. The 12 experiments consisted of four factors (fungi propagation, cutting, dispersion and incorporation), which were randomly assigned to the experimental plots.

An L _12 _orthogonal array design was used in the experiment ( Taguchi, 1986). This design was used to ensure robust experimental results under a wide range of conditions. Only the main effects were seen in the experiments, and all the factor levels had six replications. The 12 experiments consisted of four factors (fungi propagation, cutting, dispersion and incorporation) that were randomly assigned to the experimental plots ( Table 1). The four different treatments were established as follows. In the fungi propagation factor, the residue was stored in a compost house for 7 days until propagating fungi, and the remainder of the residue stored beside the field. For the cutting factor, the residue was cut into 10 cm pieces. For the dispersion factor, the residue was scattered evenly on the plots, and for the non-dispersion, the residue was placed on the center line of the plot in a swath approximately 30 cm wide. For the incorporation factor, I removed approximately 5 cm thickness of soil of the plots, then placed the residue, and covered the residue with the soil ( Figure 1).

**Table 1.** Experimental matrix for L _12_ orthogonal array. The 12 experiments consisted of four factors (fungi propagation, cutting, dispersion and incorporation), which were randomly assigned to the experimental plots.

No.	Fungi propagation	Cutting	Dispersion	Incorporation
1	◯		◯	
2				◯
3	◯	◯		
4	◯			◯
5			◯	◯
6	◯	◯	◯	◯
7		◯	◯	
8	◯			
9		◯		◯
10		◯		
11	◯	◯	◯	◯
12			◯	

### Validation of the linearity of input and output

Strong linearity must be found between returned residue quantity and the yield if the adopted factors have a robust performance ( Taguchi, 1986). For validation purposes, I returned the crop residues into the same plot using the combination of the two most effective factors (dispersion and incorporation). The plot effect can confound the yields in this plot design since the quantity (0.4–2.0 Mg ha ^−1^ in dry weight) of applied residues was proportional to the preceding yield in each plot. However, this conflict was considered to be small because the plot effect was very small in this field (see raw data ( Oda, 2018)) and the after effect of corn was small. I seeded corn on October 28, 2014 and harvested it on January 9, 2015 (73 days after seeding).

### Determination of yields and statistical analyses

I got precipitation data from the automatic weather station at the Laura Farm. I weighed the fresh weight of the whole crop and the kernels in each plot and checked the kernel/whole ratio was stable ( *R* ^2^ = 0.9983). The aboveground dry matter (DM) was calculated by multiplying the fresh weight to an aboveground DM/fresh-kernel-weight ratio (2.79) of an existing study ( Miura & Watanabe, 2002). The aboveground residue DM was calculated by multiplying the DM/fresh ratio (0.46) of air-dried residue sample. Statistical Effect size analysis was conducted using the effect size of Cohen’s *d* ( Cohen, 1992) by the MS Excel 2016.

$$\text{d}=\frac{M_{\text{1}}-M_{\text{2}}}{\sqrt{\frac{SD_{\text{1}}^{\text{2}}+SD_{\text{2}}^{\text{2}}}{2}}}$$

d: Effect size, M: Mean, SD: Standard deviation

A significance is affected by the sample size; however, Cohen's *d* is not affected by sample sizes and can evaluate the true effect. The *p* values were calculated with an unpaired, one-sided, unequal variances *t*-test. I evaluated the linearity of the input and the output using the coefficient of determination ( *R* ^2^) in a simple linear regression.

## Results

### Climate conditions

There were no irregular climate conditions during the experimental periods. The number of precipitation days for each 78-day and 73-day crop period were 58 and 54 days, respectively, and total precipitation was 1111 and 542 mm, respectively. Based on the amount of undecomposed residue collected, the decomposition rate of the input residue was nearly the same for incorporation (90%) and non-incorporation (85%).

### Effect size of the application methods

Table 2 shows the results of the Effect size analyses. The effect sizes of Cohen’s *d* < 0.2, 0.5, 0.8 and 1.2, and *d* > 2.0 correspond to small, medium, large, very large and huge, respectively ( Cohen, 1992; Sawilowsky, 2009).

**Table 2.** Effect size of crop residue application method factors on crop yield. The same amount of crop residue was applied to plots by different methods (fungi propagation, cutting, dispersion and incorporation).

Factor	Cohen’s d	P
Fungi propagation	0.1	0.455
Cutting	0.0	0.477
Dispersion	1.2	0.045
Incorporation	0.7	0.223

P values: one-sided, unpaired, unequal distribution t-test (n = 6).

Dispersion had a larger effect ( *p* = 0.045, Cohen’s *d* = 1.2) than incorporation ( *p* = 0.223, Cohen’s *d* = 0.7). Fungi propagation and cutting had no effect (Cohen’s *d* = 0.0–0.1). I calculated the effects of dispersion and incorporation, assuming the effects of cutting and fungi propagation to be negligible. The interaction was huge ( *p* = 0.005, Cohen’s *d* = 4.9), while the main effect of dispersion (Cohen’s *d* = 0.3) and incorporation (Cohen’s d = −0.2) were medium and small in effect size, respectively ( Table 3).

**Table 3.** Estimated effect size of dispersion and incorporating. The same amount of crop residue was applied to plots by different methods (fungi propagation, cutting, dispersion and incorporation). The effect sizes on corn yields were calculated neglecting the un-effective factors in Table 2. P values: one-sided, unpaired, unequal distribution t-test (n = 3).

Factor	Cohen’s d	p	
Dispersion	Incorporation		
+	+	4.9	0.005
+	–	0.3	0.392
–	+	–0.2	0.420

### Linearity of input and output

**Figure 2.**
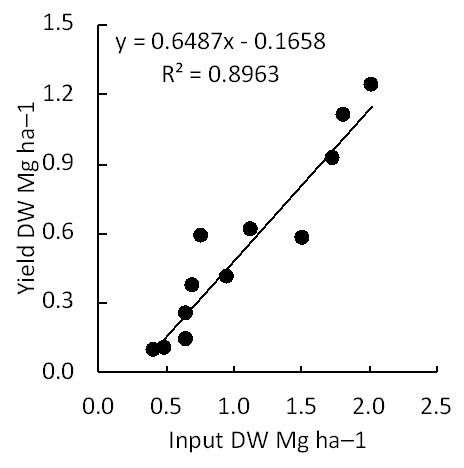
Biomass output based on the quantity of corn residue input. Crop residues were returned into the same plot using the combination of the two most effective factors (dispersion and incorporation).

Under the combination of dispersion and incorporation, input (the quantity of applied residue) was proportional to the output (harvested aboveground biomass) ( Figure 2; *R* ^2^ = 0.8963).

## Discussion

### Effect of dispersion

Dispersion had a very large effect on corn yield. The yield was influenced by the huge effect of the interaction of dispersion and incorporation. The dispersion of crop residue application having a large effect on yield is probably a new finding. The C/N ratio of sweet corn at harvest is generally 30 to 35, which can cause nitrogen starvation.

A positive effect for dispersion is surprising, given that dispersion typically increases nutrient loss. Intuitively, application of crop residues away from plants should be less effective ( Stone *et al*., 2000). The results of this study will be reasonable if dispersion avoids the negative effect of residue clustering. This is because crop residue retained at the surface or incorporated into the soil may produce phytotoxic allelochemicals ( Chung & Miller, 1995; Weston, 1996). In addition, Martin *et al*. (1990) reported that corn stover is itself phytotoxic. The combination of the toxicity produced by the decomposition of residue by bacteria, fungi and extract of residue results in different levels of toxicity.

For the aspect of adsorption, dispersion increases the adsorption of phytotoxic substances in soil. Adsorption is the most important soil factor controlling the fate of chemicals in the environment because it controls the chemical concentrations present in the soil solution, which is a physiological phenomenon that follows the Freundlich equation ( Vidal & Bauman, 1997). This means that dispersion has a stable effect on avoiding phytotoxicity. We should pay closer attention to the dispersion of residue incorporation because tillage merely buries the residue ( Staricka *et al*., 1991).

### Synergistic effect of dispersion and incorporation

Conversely, the positive effect of incorporating crop residue on yield is because of the nutrient addition ( Kumar & Goh, 1999), especially given that this experiment was conducted without fertilizer addition. Matching the amount and timing of nitrogen release with crop nitrogen demand is a key component of crop production ( Robertson, 1997).

The combination of dispersion and incorporation brings a proportional yield increase to applied residue under non-fertilized conditions. In this case, the yield will reflect the nutrient applied by the residue. Furthermore, incorporation had an interactive effect, but surface placement did not. Residue incorporation, rather than surface placement, may enhance biological nitrogen fixation by reducing the inorganic N in soil ( Kumar & Goh, 1999); however, further study is needed in this area.

## Conclusion

A stable climate with a low after effect of soil can reveal a stable result for crop residue management. Cohen's effect size analysis showed the dispersion of crop residue had a very large effect on corn yield, and the effect arose from the interaction with incorporation. Dispersion will avoid phytotoxicity from anaerobic conditions inside residue clusters and clarify the positive effect of crop residue incorporation. This finding adding a new viewpoint for the controversy between conventional and conservation agriculture.

## Ethics statement

This study was conducted with the permissions from the local government.

## Data Availability

Figshare: Majuro Supplemental data, https://doi.org/10.6084/m9.figshare.6668129.v1 ( Oda, 2018). Data from Experiments 3 and 4 includes data that supports the results of this article. Data are available under the terms of the Creative Commons Zero "No rights reserved" data waiver (CC0 1.0 Public domain dedication).
