# Supplementary material for: Dispersion is essential in crop residue application
Source: F1000Res. 2025 Dec 16;7:1831. Originally published 2018 Nov 21. [Version 4] doi: 10.12688/f1000research.16748.4 (PMC12511857; doi:10.12688/f1000research.16748.4)
Supplement: Peer review history

# Reviewer report: Reviewer response for version 3

**DOI:** 10.5256/f1000research.187288.r414941

**Published:** 2025-10-09

**Authors:** Zachary Brym

**Affiliations:** 1 University of Florida, Homestead, Florida, USA

This article succinctly presents an experiment that investigates residue application factors in a unique environment. The study and its reporting is interesting and of potential importance. In the interest of a detailed and constructive review, I have provided several comments for consideration and clarification. These included suggestions on literature to incorporate and expansions to the text to help the reader understand the derivation and context for important details.

 Abstract

 Background

 - The statement "effects of residue application..." is unclear. What would make the effects "stable"? What is a "residual effect" related to crop residue. Some of the confusion is by the overlapping definitions of these similar words.

 Methods

 - This is an intriguing residue evaluation and experimental design. 

 Results

 - A bit more information on the dispersion method would help the interpretation of results here.

 Discussion

 - How was toxicity determined for the corn residue? What is the source of toxicity by corn residue? Is it nitrate, organic acid, etc.? 

 Introduction

 - I suggest you provide an extra detail about which substances of microorganisms are toxic.

 - A recent review of microbial degradation of residue (https://link.springer.com/article/10.1007/s13762-024-06209-z) to consider adding.

 - In counter point to lacking evidence, this review acknowledged several long-term studies on crop residue effects (https://doi.org/10.1080/21683565.2017.1336149).

 Method

 - I appreciate the intent to use a uniquely stable environment for the experiment.

 - What is the effect of multi-year corn crop on the site location? What is the C:N ratio of the harvested corn? 

 - Reporting is just a single year study, though two crop cycles are considered separately for study objectives.

 Results

 -The results are simple and adequate. Combination of dispersion and incorporation makes sense for moderate material decomposition, based on the observation that most material was decomposed by crop harvest. 

 - Was there variability in weed pressure observed across the plots? Thinking about ways dispersion might impact soil characteristics and crop growth.

 Discussion

 - "probably a new finding" seems like an unnecessary statement. Your intuition is to place residue at the base of plants, but my understanding is that the roots will be distributed laterally across the row. My intuition is to distribute the residue evenly across the field, if not for positive residue effect specifically, then for consistent effect across a field.  Is there a published recommendation for placing on center line? Here's a review on root architecture (https://doi.org/10.1093/aob/mcs293)

 - Interesting residue toxicity is indicated as the primary driver above organic matter and nutrient retention. I struggle a bit to reconcile the factor of crop residue organic matter, nutrition, and phytotoxicity. In the soils of this study, overall how do crop plants respond? What is the nutrient balance of the soil as it relates to crop productivity? What aspects of the study lead you to identify toxicity as a key driver?

Is the work clearly and accurately presented and does it cite the current literature?

Partly

If applicable, is the statistical analysis and its interpretation appropriate?

Yes

Are all the source data underlying the results available to ensure full reproducibility?

Yes

Is the study design appropriate and is the work technically sound?

Yes

Are the conclusions drawn adequately supported by the results?

Yes

Are sufficient details of methods and analysis provided to allow replication by others?

Yes

Reviewer Expertise:

Agroecology, Agronomy, Best Management Practices

I confirm that I have read this submission and believe that I have an appropriate level of expertise to confirm that it is of an acceptable scientific standard.

# Reviewer report: Reviewer response for version 2

**DOI:** 10.5256/f1000research.24786.r170344

**Published:** 2024-02-07

**Authors:** Valensi Kautsar

**Affiliations:** 1 Institut Pertanian Stiper, Instiper Yogyakarta, Yogyakarta, Indonesia

1. Which groups are used to calculate Cohen’s d value? For example, to analyze the dispersion effect, the group of dispersion treatment is compared to which group for control?
2. In the Methods: "all factor levels have six replicates". Does the replication mean the number of plant replicates in one plot?

Is the work clearly and accurately presented and does it cite the current literature?

Yes

If applicable, is the statistical analysis and its interpretation appropriate?

Yes

Are all the source data underlying the results available to ensure full reproducibility?

Yes

Is the study design appropriate and is the work technically sound?

Yes

Are the conclusions drawn adequately supported by the results?

Yes

Are sufficient details of methods and analysis provided to allow replication by others?

Yes

Reviewer Expertise:

internal nutrient cycling

I confirm that I have read this submission and believe that I have an appropriate level of expertise to confirm that it is of an acceptable scientific standard.

# Response

**Published:** 2024-03-05

**Authors:** Masato Oda

**Affiliations:** Independent Scientist, Japan

Thank you for your precious comment. I'm sorry to be late to reply.

 The answer for your questions are as follows.

**1. Which groups are used to calculate Cohen’s d value?**

 For each treatment.

 Every treatment has 6 replication plots. The effect of each treatment is assumed as an additive effect. 

 This study design cannot detect interactions. An interaction can be detected if the number of treatments is 2 instead of 4. So, I assumed that the study was designed for two treatments (dispersion and incorporation) because the Cohen's d for cutting or fungal propagation were very small (less than 0.1).

**2. Does the replication mean the number of plant replicates in one plot?**

 No.

 The replication means the number of plots. Factorial design experiment reduces the number of experimental plots by limiting the detection of interactions.

# Response

**Published:** 2024-06-04

**Authors:** Masato Oda

**Affiliations:** Independent Scientist, Japan

Thanks for the review. Sorry for the late response.

1. **Which groups are used to calculate Cohen’s d value?**
  This experiment is designed using an orthogonal array, so the effect of each treatment is compared to  the overall mean value.
2. **Does the replication mean the number of plant replicates in one plot?**
  No. The number of replicates is the number of plots.

The Taguchi method is a highly efficient experimental design. It is widely used in industry.
